# Exploring Mental Health Content Moderation and Well-Being Tools on Social Media Platforms: Walkthrough Analysis

**DOI:** 10.2196/69817

**Published:** 2025-05-29

**Authors:** Zoë Haime, Lucy Biddle

**Affiliations:** 1 Population Health Sciences University of Bristol Bristol United Kingdom; 2 NIHR Bristol Biomedical Research Centre Bristol United Kingdom; 3 NIHR Applied Research Collaboration West Bristol United Kingdom

**Keywords:** online moderation, mental health, user experience, social networking, online well-being

## Abstract

**Background:**

Social networking site (SNS) users may experience mental health difficulties themselves or engage with mental health–related content on these platforms. While SNSs use moderation systems and user tools to limit harmful content availability, concerns persist regarding the implementation and effectiveness of these methods.

**Objective:**

This study aimed to use an ethnographic walkthrough method to critically evaluate 4 SNSs—Instagram, TikTok, Tumblr, and Tellmi.

**Methods:**

Walkthrough methods were used to identify and analyze mental health content moderation and safety and well-being resources of SNS platforms. We completed systematic checklists for each of the SNS platforms and then used thematic analysis to interpret the data.

**Results:**

Findings highlighted both successes and challenges in balancing user safety and content moderation across platforms. While varied mental health resources were available on platforms, several issues emerged, including redundancy of information, broken links, and a lack of non–US-centric resources. In addition, despite the presence of several self-moderation tool options, there was insufficient evidence of user education and testing around these features, potentially limiting their effectiveness. Platforms also faced difficulties addressing harmful mental health content due to unclear language around what was allowed or disallowed. This was especially evident in the management of mental health–related terminology, where the emergence of “algospeak,” where users adopt alternative codewords or phrases to avoid having content removed or banned by moderation systems, highlighted how users easily bypass platform censorship. Furthermore, platforms did not detail support for reporters or reportees of mental health–related content, leaving users susceptible.

**Conclusions:**

Our study resulted in the production of preliminary recommendations for platforms regarding potential mental health content moderation and well-being procedures and tools. We also emphasized the need for more inclusive user-centered design, feedback, and research to improve SNS safety and moderation features.

## Introduction

### Background

Social networking sites (SNSs) can serve multiple purposes for individuals with mental health difficulties [1]. These include facilitating self-disclosure of mental health disorders, acting as discursive spaces, and offering exposure to both harmful and helpful mental health–related content [2,3]. However, several research studies have identified relationships between SNS use and development or exacerbation of mental health symptoms, including depression, anxiety, psychosis, eating disorder, self-harm, and suicide behaviors [4,5].

With approximately 1 in 8 people globally living with a mental health condition [6] and considering the multifaceted role SNSs can have in shaping mental health experiences, platforms have a significant responsibility to support and guide users in managing their well-being. SNSs currently use a variety of moderation and safety techniques to regulate their online communities, including blocking and removing content, banning users, outlining community guidelines, and providing mental health resources, through automatic and manual processes [7].

Despite this, concerns remain about the effectiveness of SNS safety practices [8]. For instance, users engaging with self-harm and suicide material online have reported actively avoiding online help during crises, including dismissing pop-ups and support links [9]. Furthermore, SNS users engaging with self-harm and suicide content online have criticized platform inconsistencies regarding content removal and the communication of decisions around this, reporting that these moderation actions can lead to feelings of stigmatization and harm [8,10]. In addition, studies have found that due to strict censorship of mental health–related terms, platform users may turn to “algospeak,” where they adopt alternative codewords or phrases to avoid having content removed or banned by moderation systems [11], or to circumnavigate search filters [12,13]. Another study, which explored how people use platforms and technology to support their mental health [14] found that users wanted the provision of mental health support on SNSs to be more nuanced and trustworthy, with clear, evidence-based guidance. Concerns have also been raised more generally about users’ ability to read and understand SNS policies and guidance. One study identified significant variation between community guidelines across platforms, highlighting how this can negatively impact user safety and trust [15]. Another study stated that users’ contract requirements with SNSs whereby they state they have read and agreed to the terms of service (ToS) is “the biggest lie on the internet” [16].

These findings highlight possible inefficiencies in current moderation and safety mechanisms used by SNSs and significant potential risks, particularly for vulnerable users, including exposure to harmful content, inadequate crisis responses, and possible disengagement from regulated spaces in favor of less-moderated ones. The full extent of such moderation and safety challenges across SNSs remains unclear, highlighting the need for more in-depth explorations of user experiences. The introduction of the Online Safety Act (OSA) [17] in the United Kingdom, which aims to tighten safety regulations and standards for digital platforms, further emphasizes this need for comprehensive data on current SNS practices and their effectiveness, to fully assess and address issues.

The walkthrough method is a valuable tool for understanding user experiences on these online platforms [18,19]. It is a structured ethnographic approach examining application and platform features, settings, and interactions by “touring” the platform interactively. The method focuses on understanding how platform design and resources potentially encourage and impact user behavior. For instance, in their paper on a walkthrough of the dating platform Tinder, Gillett [20] identified several user safety issues, including the problematic placement and design of the reporting icon. The author proposed that these design choices could significantly influence user behavior, leading to potential unintended interactions with harmful content and adverse effects on user well-being. This study demonstrated how the walkthrough method can be used to reveal critical aspects of user experience that might otherwise be overlooked.

### This Study

Using the walkthrough method, this study aimed to systematically identify how SNSs manage mental health content and user well-being through moderation, safety features, resources, and policies and to explore the potential impact of these platform attributes on users. By identifying platform successes and areas for improvement, the study aimed to contribute to the creation of safer and more supportive digital environments.

## Methods

### Research Design

This study used an ethnographic walkthrough method to systematically and critically assess the approaches of various SNSs in moderating mental health content and promoting user well-being. Researchers actively participated in the platforms as observers to evaluate their features, guidance, and resources.

### Ethical Considerations

This study adhered to ethical guidelines for internet-mediated research [21]. Researchers ensured that their participation did not interfere with the functioning of the platforms, mislead other users, or go against platform regulations. Confidentiality was maintained by avoiding the recording of personally identifiable information, such as user-generated names or specific images. No interventions were made in response to harmful content observed, ensuring that the user experience and platform responses remained unaltered. No financial compensation was provided to researchers as they undertook the research as part of their routine professional role. There were well-being procedures in place for researchers, and they were able to withdraw from conducting the walkthrough checklists if they wished.

Ethics approval was received from the Faculty of Health Sciences Research Ethics Committee at the University of Bristol (approval number 17972).

### Positionality Statement

As researchers in the field of mental health and digital technology, we acknowledge that our personal and professional experiences shape our perspectives and approach to this study. Researchers ZH and LB are both White women from the United Kingdom. ZH identifies as a digital native, reflecting a comfort with digital technology and early adoption of various online platforms throughout her life. In contrast, LB is a digital immigrant.

### Data Collection

We selected 4 SNS platforms for evaluation. TikTok and Instagram were selected due to their status as two of the most widely used social media platforms in the United Kingdom, particularly among adolescents [22,23]. Tumblr was chosen for its established reputation as a significant SNS platform, offering a contrasting perspective to the newer platforms. In addition, although Tumblr has less representation in the current research literature and discussion around online safety [23], media reports indicate a recent shift in their user base toward the Gen Z population [24,25]. Tellmi was chosen as a smaller platform specializing in mental health support and discussion among young people, which offered an opportunity to evaluate a platform specifically designed with the mental health of users in mind, providing a useful comparison to SNSs that prioritize broader aspects of social interaction. For this study, Facebook was excluded from our SNS evaluation despite it being the most accessed platform in the United Kingdom. This was because of its lower popularity among younger UK users [23] and its likely similarities with Instagram, given their shared ownership by Meta.

To conduct our walkthroughs, we used 2 checklists (Multimedia Appendices 1 and 2) to systematically evaluate each platform’s approach to moderating mental health content and supporting user well-being. Checklists were developed from previous research findings [8,26] and industry guidelines for suicide and self-harm content [27]. To conduct the walkthrough checklist, ZH used a new iOS device and LB used a web browser. Table 1 depicts the platform areas that the checklist focused on, and Table 2 shows the stages of use that were observed and documented through field notes and screenshots.

**Table 1:** Checklist focus areas.

Checklist focus areas	Description
Design, language, and features	Assessment of the design, language, and features of platform content related to mental health, safety, and well-being.
Policies and processes	Evaluation of the accessibility, availability, and relevance of information and policies concerning age limits, mental health rules, and safety guidelines.
Resources	Analysis of the accessibility, availability, and relevance of mental health resources, including those tailored for specific populations such as young people and parents or guardians.
Moderation processes	Examination of moderation processes, including self-moderation tools.

**Table 2:** Documented platform stages of use.

Stages of use	Details
Sign-up	For sign-up purposes, researchers entered a date of birth, making the account user <13 years, 15 years, and 19 years old, respectively. No account was created to comply with platform policies. New accounts were created by the researcher using their own date of birth for checklist completion. Tellmi also required users to enter a UK postcode. Researchers entered their own UK postcode. No other demographic data (eg, gender, ethnicity, and sexual orientation) was provided by researchers.
Standard platform use	Observations during typical use of the platform environment. Researchers only observed content and did not engage (eg, like, share, or comment) with content during this phase. However, in the case of Tumblr, some interactions (eg, following hashtags) were required during the sign-up process to proceed, as detailed in the Results section.
Searching for mental health terms	Searches were conducted for mental health–related terms, including algospeak terms related to eating disorders, self-harm, and suicide. Algospeak terms were informed by the Digital Dialogues Young Person’s Group [28]. Algospeak terms are not listed to avoid unintentionally increasing their visibility. The full list is available to researchers on request to the authors.
Reporting mental health content	Reporting processes were observed, but no content or user reports were submitted to the platform.
Exploration of moderation, safety, and well-being information	Information provided by platforms about moderation, safety, and well-being was observed.
Exploration of self-moderation tools	Self-moderation tools available on platforms were observed and used.

### Data Analysis

Data were analyzed using a thematic analysis approach [29,30]. Initially, ZH reviewed the field notes, observations, and screenshots, applying a deductive approach to generating codes based on the checklist format. These codes were then further refined through an iterative process. The codes were then organized into groups, based on similarities in the issues, ideas, or patterns they addressed. These grouped codes were then defined as themes that represented meaningful constructs of the coded data. Data for each theme can be viewed in a comparative table, by platform (Multimedia Appendix 3).

## Results

### Overview

Data were analyzed to understand the current provisions by platforms regarding mental health–related content moderation and user well-being. The themes we defined are summarized in Table 3.

**Table 3:** Theme and theme descriptions.

Theme	Theme description
Observations of safety features	Includes how users are introduced to safety on the platform, particularly through explicit safety instructions during the user journey, and moderation interventions, such as during searches for mental health–related terms
Platform moderation and safety guidance and systems	Examines how platforms describe the mechanisms for content moderation, including automatic filters, content warnings, and moderation policies that impact what users see and how content is managed
User “self-moderation” tools and reporting	Explores functionality, accessibility, and availability of self-moderation tools for users
Platform support and resources	Explores the information and resources provided by platforms related to mental health and well-being
Platform interface and communication	Explores the style, format, and presentation of mental health–related information and tools to users via the platform

### Observation of Safety Features

#### Sign-Up Processes

Across platforms, sign-up procedures generally lacked emphasis on key safety information. Instagram, TikTok, and Tumblr gave subtle displays of their ToS and privacy policies, without proactively engaging users with them. In addition, at sign-up, no platform provided direct links to their community guidelines (Moderation Guidelines on Tellmi), which detail important mental health content moderation information. Tellmi provided more safety-related prompts during the sign-up process, ensuring users agreed to data-sharing practices and were aware of the platform’s limitations in handling mental health crises. In addition, they directed users to the ToS, making it clear that this was a key part of their agreement with the platform.

Age requirements varied, Instagram and TikTok set the minimum at 13 years, Tellmi set the minimum at 11 years, and Tumblr set the minimum at 16 years. During sign-up, TikTok and Instagram restricted users from changing their birthdate if they had originally attempted to sign up below the minimum age, while Tumblr and Tellmi allowed users to enter a different date and continue, exposing a lack of age verification procedures. Notably, during sign-up, TikTok offered additional videos to users aged 13 to 17 years on data protection and default settings, reflecting an attempt to mitigate risks for younger users.

As part of the sign-up process, Tumblr required users to follow other accounts before accessing content, by selecting from the platform’s recommendation system for popular hashtags and accounts. We selected recommended hashtags that we believed could be problematic, which exposed us to potentially harmful disordered eating content early on. This highlighted insufficient moderation within Tumblr’s algorithm-driven suggestions. In contrast, Instagram also directed users to engage with popular content, but all suggestions were verified, and we did not identify any potentially problematic accounts. Users also had the option to skip this step. TikTok and Tellmi did not prompt users to follow any specific content tags or accounts during sign-up.

#### Content Exposure

Initial platform use provided varying experiences in terms of content exposure. Tellmi, Instagram, and TikTok took deliberate steps to provide users with safe, moderated content. Tellmi presented users with a wide range of nonharmful posts on mental health–related topics, while Instagram and TikTok’s algorithms effectively prioritized popular content such as humor, sports, and celebrities, avoiding explicitly harmful material. In contrast, due to Tumblr’s requirements to follow accounts and the potentially problematic ones we chose, we were immediately exposed to harmful mental health–related content in our feed including images of graphic self-harm and severely underweight female-presenting figures, as well as further recommendations to follow other seemingly harmful accounts. In addition, other than during the sign-up of users aged 13 to 17 years on TikTok, where some self-moderation tools were mentioned in videos, none of the platforms offered explicit guidance on how to find or implement self-moderation or safety tools during initial use.

#### Mental Health–Related Content Searches

When searching for mental health–related terms, using default sensitive or mature content levels, the platforms differed in their handling of material. Tellmi allowed search results for discussions on topics such as suicide, self-harm, and eating disorders, but the content was moderated and nonharmful, focusing on recovery and support. In comparison, the same suicide and self-harm term searches returned no results on Instagram, TikTok, or Tumblr, although all 3 offered relevant support. Instagram provided details for multiple external support organizations, and their own general support resource on self-care tips. TikTok gave details for Samaritans, as well as their own support resource on self-harm and suicide, and Tumblr provided details for mainly US-based organizations, as well as their internal “counseling and prevention” page. Neither Instagram nor TikTok is linked to their more developed internal self-harm and suicide help pages, available in their platform safety centers. For searches on the terms “eating disorders,” “anorexia,” and “bulimia,” Tumblr showed a similar help message with details for several more US organizations and no results. TikTok showed no results but directed users to its internal “eating disorders” resource and contact details for Beat An Instagram search for “eating disorders” gave the same resource page as for “suicide” and “self-harm” searches on the platform. For the terms “anorexia” and “bulimia,” Instagram gave these resources again but also provided the option for users to continue their search. Results included recovery and support posts, images of self-harm scars, content promoting disordered eating, related potentially harmful hashtags, access to user biographies that stated goal weights (below healthy adult weights), and the phrase “block don’t report” (also found in Tumblr results). On Instagram, sensitive content warnings also appeared on some posts but were inconsistently applied.

In searches for algospeak, all platforms returned results for at least 1 algospeak term related to self-harm, suicide, or eating disorders. On Instagram, TikTok, and Tumblr, algospeak terms led to more graphic and harmful content, including identification of further algospeak hashtags. Tellmi again returned moderated content, meaning the discussions presented were not harmful.

### Platform Moderation and Safety Guidance and Systems

#### Moderation Transparency

All platforms provided information on the moderation of mental health content, primarily through their community guidelines or equivalent. The level of detail and transparency varied, with Instagram and TikTok offering extensive information on mental health content moderation and user well-being across various internally dedicated “centers,” “guides,” and “resources.” However, this spread of information often led to repetition, required users to navigate multiple links, and sometimes failed to provide essential resources for help and support such as details of emergency or external support services. Tumblr and Tellmi provided limited internal spaces for users to access detailed information when encountering specific mental health–related content or seeking support for their own mental health needs. Users on Tellmi could access relevant areas of the directory, and on Tumblr, their “counselling and prevention resources” page. However, in both cases, resources were external to the platform.

**Figure 1:**
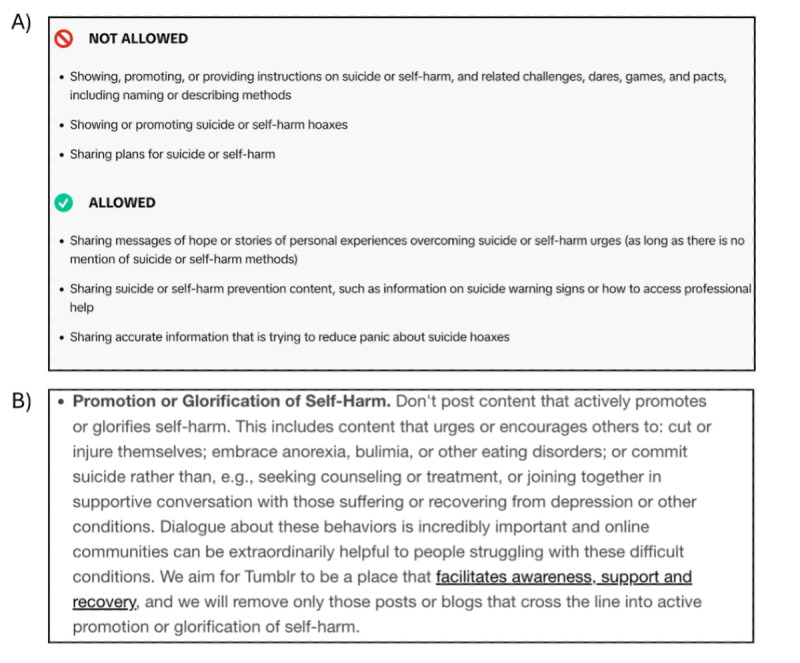
(A) Screenshot of TikTok community guidelines for allowed and disallowed suicide and self-harm content. (B) Screenshot of Tumblr user guidelines for promotion or glorification of self-harm.

TikTok and Instagram provided the most concise and specific information about what mental health–related content was and was not allowed on their platform, including clear examples. Instagram further outlined content they may label as sensitive, such as “older instances of self-harm such as healed cuts” or “content that depicts ribs, collar bones, thigh gaps, hips, concave stomach or protruding spine or scapula in a recovery context.” In contrast, Tumblr and Tellmi guidance offered less clarity regarding disallowed content, often merging eating disorder, self-harm, or suicide content into a single category and using more abstract language, such as Tumblr prohibiting content that “embrace[s] anorexia” (Figure 1).

#### Content Moderation Systems and Enforcement

TikTok, Tumblr, and Instagram described the use of automated moderation systems alongside human moderators, although roles were sometimes unclear. TikTok and Instagram further provided quarterly updates through their transparency centers, offering insights into the volume of mental health–related content removed both automatically and through user reporting. Tellmi detailed their unique approach of using entirely human moderation, emphasizing how this allowed for more nuanced decision-making and a personal approach to safety. However, we noted Tellmi moderators went offline from 11 PM to 8:30 AM (UK time), meaning no posting, and we also noted that during periods of heavy user engagement, posting would also be restricted to manage moderation load.

All platforms indicated that they could ban users or remove their content if it did not meet their required standards. However, the mechanisms and systems for enforcing these rules varied significantly. For instance, TikTok used a “strike” system to manage violations, but the details were somewhat vague, as the specifics of how strikes were issued and accumulated were not clearly communicated. Instagram stated that users “may” receive warnings before content was removed; however, the language used was noncommittal, leading to ambiguity about the enforcement process. TikTok and Instagram did allow users to track their account status, which indicated violations to the user and allowed for efficient use of the appeals process. Alternatively, Tumblr stated that they would inform users only after removing content or terminating accounts, except in the case of sexually explicit content, where users were given an opportunity to review, amend, or remove content. Tellmi’s approach was more explicit, stating that posts or replies that did not meet their moderation standards would be rejected and users would receive a brief explanation for the rejection to which they could directly respond to a human moderator.

#### Age-Based Protections

TikTok and, more recently, Instagram have implemented age-related defaults aimed at protecting young users, particularly those aged between 13 and 17 years. These focus on limiting potentially sensitive content exposure, managing screen time, and restricting interactions with unknown users. Both platforms enforce age-related settings with distinct approaches. On TikTok, younger teenagers (aged 13-15 years) face restrictions that cannot be altered. These include preventing others from downloading their videos, blocking all direct messaging, keeping accounts private, and disabling features such as video stitching, duetting, and comments (stitching refers to combining an existing video with one you are creating; duetting refers to you posting your video side-by-side with an existing video on TikTok). Instagram similarly defaults those aged 13 to 15 years to private accounts and enforces restrictions including time limits, sleep mode, and disabling interaction features. Unlike TikTok, Instagram allows teenagers to request changes to these settings, but parental approval is required for modifications. Teenagers aged 16 to 17 years on both platforms have slightly more flexibility, such as enabling public accounts, but on Instagram, parental supervision is still necessary for approving changes. Alternatively, Tellmi adopted an age-banding approach to ensure users <20 years primarily interact with peers within 2 years of their age. Despite this, Tellmi informs users that responses to discussions may introduce wider age gaps, potentially affecting their intended interactions. In addition, Tumblr limits potentially mature content to audiences aged >18 years but does not implement any other age-based functions.

### User “Self-Moderation” Tools and Reporting

#### User Control and Access to Tools

Self-moderation tools across platforms were often presented as options in user profile menus but with limited context regarding their potential effects. Even where their functionality was described in detail on platform help or safety pages, it was rare that well-being benefits were reflected to the user. Instagram and TikTok provided 21 and 23 self-moderation tools, respectively, offering time management, filtering, and privacy controls. Generally, these tools were in similar places on the platforms, making them intuitive to locate for those familiar with these types of online environments, but occasionally, they were in unexpected spaces or were less straightforward to implement. Some tools seemed to have overlapping functions, which may inhibit users’ understanding of their functionality. Time management tools offered by Instagram and TikTok included prompts to take screen breaks, have quiet time, or go to sleep. These were easily accessible in time management controls of the respective platforms, where users could also view a visual tracker of their engagement. Both platforms offered preset time options for these tools, with TikTok’s settings being slightly more customizable than Instagram’s. However, the imposed limits on these presets suggested that neither platform was actively promoting extended or frequent disengagement. Self-moderation tools on the other platforms were much more limited. Tumblr offered 12 tools, primarily focused on privacy with some options for content filtering, and while Tellmi’s role as a mental health support platform may make disengagement tools less intuitive, they provided 3 relevant self-moderation tools, with only 1 for hiding content related to certain mental health categories, limiting user control over their experiences.

All platforms also implemented some form of sensitivity content rating, mostly to prevent access to what they termed mature content. TikTok, Tumblr, and Instagram automatically restricted such content for users aged <18 years, although definitions of mature or sensitive content for TikTok and Tumblr were vague. Tellmi offered greater transparency by clearly indicating that higher sensitivity would limit access to potentially triggering content. However, the specific content covered by each sensitivity level remained unclear. On Instagram and Tellmi, where sensitivity ratings could be adjusted by users, more restrictive settings limited search results for mental health and algospeak terms, suggesting that mental health content was included in these sensitivity categories for both platforms.

#### Reporting Systems

**Figure 2:**
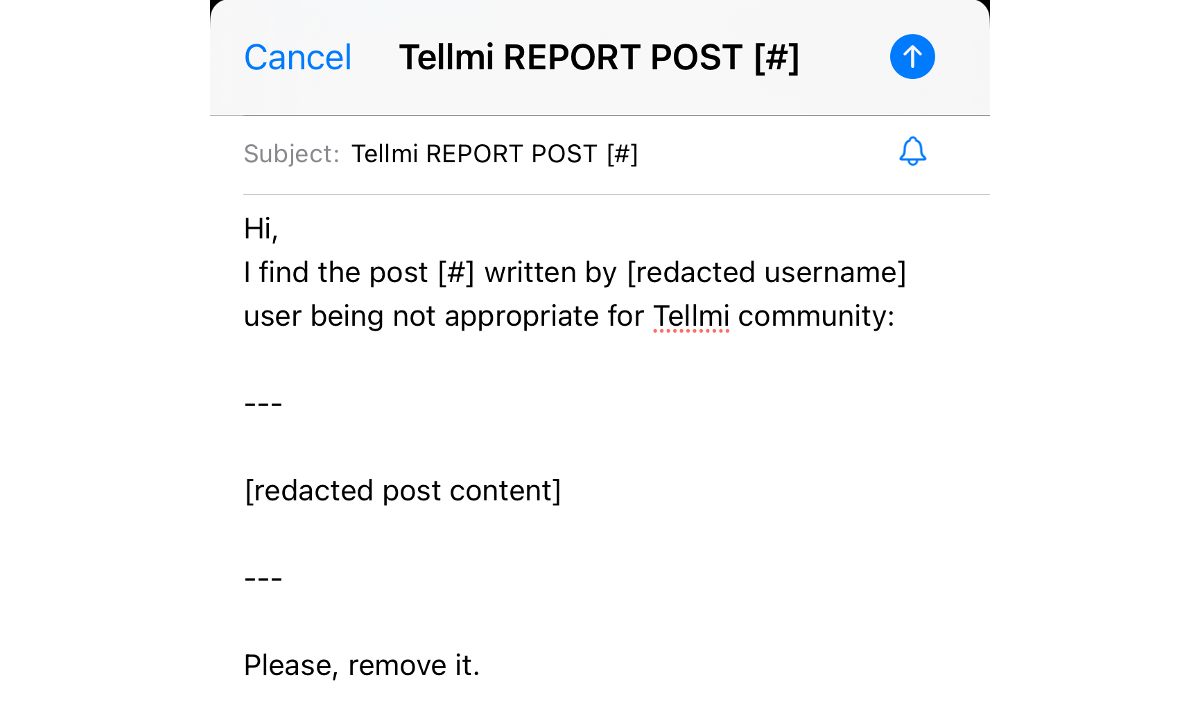
Screenshot of an automated Tellmi template report email.

All platforms also shared similarities in encouraging users to report harmful content throughout their health, safety, and policy pages, often referring to user responsibility to report others’ content that violates platform terms. Instagram and TikTok allowed users to report specific categories of mental health content such as suicide, self-harm, and disordered eating. Tumblr only provided a mental health content option for reporting suicide and self-harm, potentially hindering users aiming to report disordered eating content. However, Tumblr’s reporting process was more detailed, requiring users to indicate if the person posting content was actively threatening suicide and to explain their concerns. In contrast, TikTok included reminders of platform rules regarding mental health–related content before users submitted their report, reinforcing guidelines and giving users a clear understanding of the platform’s expectations. Tellmi offered a more manual process, where users reported any content by sending an email to the platform’s support team via an automated template that could be personalized before submission (Figure 2). This method contrasted with the more automated systems of the other platforms.

The platforms also differed in how they handled report follow-ups. Instagram and TikTok allowed users to check the status of their reports and appeal any decisions made, creating a straightforward process for feedback. Notably, Instagram also indicated that user reports may influence the content algorithm for their feed:

We may use your report to make content similar to what you reported appear lower in your Feed or in the recommendations we make for you, such as Suggested posts and Explore.

Instagram’s “Staying Safe” section in the Help Centre

The other platforms lacked tracking features for their reporting systems, although Tellmi did offer email correspondence regarding decisions made on reports, allowing users to engage directly with a human moderator. None of the platforms provided information about whether they offered follow-up with support or resources for users who reported mental health–related content or for those whose content was reported, and as we did not report mental health content within this study, we cannot confirm their processes.

### Platform Support and Resources

Platform resources for mental health support varied significantly. Instagram and TikTok provided a range of internal resources on self-harm, suicide, and disordered eating, including pages with practical advice, educational content, and emergency procedures that were written in consultation with credible external organizations. Uniquely, TikTok also published a page focused on creator burnout, and Tellmi allowed users to complete and track scores in mental health quizzes. In addition, Instagram hosted a variety of resources around mental health that had been developed and published by external services and charities, which users were able to download. Similarly, Tellmi directed users to a wealth of resources hosted externally. Both Instagram and TikTok provided a somewhat overwhelmingly wide range of guidance and policy pages directed at young people and their parents or guardians, again providing information on well-being support and practical online advice. Tumblr only provided 1 brief page for users that aimed to help with mental health needs, giving specific advice on what to do in response to immediate harm. On this page, Tumblr also gave contact details for a couple of UK-based mental health organizations. Tellmi and Instagram offered a broader range of relevant organizations, including those focused on mental health, online harms, and intersectional factors such as sexuality and religion. Notably, TikTok, Instagram, and Tumblr often featured US-centric resources across help and safety pages, which sometimes excluded other countries.

### Platform Interface and Communication

Platforms generally communicated with their users using clear and professional language, suitable for their audiences. However, Tumblr’s casual style veered into potentially patronizing territory:

You have to be the Minimum Age to use Tumblr. We’re serious: it’s a hard rule. “But I’m, like, almost old enough!” you plead. Nope, sorry. If you’re not old enough, don’t use Tumblr. Ask your parents for a PlayStation 4, or try books.

Tumblr’s ToS

All platforms occasionally relied on nondefinite language regarding processes, which could potentially confuse users and create opportunities for inconsistent practices. When conveying information, Instagram and TikTok were reasonably text heavy, although attempts were made to use more mixed media in youth-centered areas of the platform. Tellmi used images and short descriptions, likely in an attempt to keep younger users engaged. Tumblr was more effective at using a mix of media—such as illustrations, GIFs, and videos—throughout their information and guidance pages aimed at all audiences.

Tellmi organized its resources and help pages in a single directory, and Tumblr, across a few pages, resulting in a concise and user-friendly experience. In contrast, Instagram and TikTok disseminated information about mental health policies, guidance, and resources across various centers, with Instagram often redirecting users to additional Meta sites as well. Although this approach offered multiple valuable resources and ways to discover them, it complicated user navigation and increased information burden and repetition.

Within the app interface, Tumblr, TikTok, and Instagram provided a trigram “hamburger” 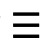 icon for access to most tools, pages, and policies, whereas Tellmi did not use familiar icons along a navigation bar. On the browser, access to all relevant documentation was available in the footer of the platform home page. In addition, several platforms functioned differently depending on device, occasionally restricting direct access to self-moderation options on browsers. Finally, Instagram’s use of hidden links—accessible only through specific paths—and TikTok and Instagram’s occasional reliance on blog posts for self-moderation tool descriptions limited accessibility for users.

## Discussion

### Principal Findings

This study extended our understanding of how a selection of SNSs manage user safety and well-being. Using walkthrough analysis, we observed both accomplishments by platforms and challenges they face in balancing content management, moderation, and user empowerment, leading to the development of preliminary recommendations for the industry (Textbox 1). Findings raised some critical concerns regarding the effectiveness of existing practices and the need for further research.

**Preliminary recommendations for the industry.**

**User onboarding and education**

- Use the first-time new user’s engagement with the platform to guide them to self-moderation tools and inform them of default settings (including those related to age restrictions) and moderation procedures.
- During sign-up, consider introducing prompts to encourage users to read the terms of service, privacy policies, and community guidelines. Alternatively, give users access to brief explanations of relevant key information from policies.
- Consider giving users the option to not follow accounts during their initial interactions on the platform.
- Consider implementing transition periods when users move into the 16- to 17-year and then >18-year age bands on age-restricted platforms. During this period, provide tailored education information on self-moderation tools and the benefits of using them.

**Reporting content or users**

- Offer clear guidance on reporting any potentially harmful mental health–related content, ensuring the process is simple and user-friendly, with any information about outreach and resources sent to reported users. Consider adding a system for users to track reports.
- Offer immediate follow-up resources to users who report mental health–related content.
- Consider a warning system for users whose content or accounts are reported for harmful mental health–related content, giving them the opportunity to review and amend their post. When content is removed or accounts are banned, ensure transparency around decisions and consider sending mental health support resources.
- Consider allowing users access to information about the effectiveness of platform moderation systems through reports on content removal and user bans for posting mental health–related content.
- Consider shifting away from language that expects users to report content and instead highlight the platform's commitment to proactive moderation and safety measures.

**Support and resources**

- Provide evidence-based internal support resources around mental health so users can access information in a familiar and trusted space. Consolidate and streamline these resources to avoid overwhelming vulnerable users.
- Ensure that resources and support are regionally and culturally relevant across platform pages. Platforms should strive to include organizations that address intersectional factors, promoting inclusivity and better serving diverse user needs.
- Consider specifying the evidence base for default safety mechanisms (including age restrictions).

**Self-moderation tools**

- Ensure users have access to a variety of self-moderation tools, offering them flexibility in managing and controlling their platform use. Clearly explain the purpose and functionality of each tool, ensuring they are distinct, practical, and easy to use.
- Ensure that users have access to clear explanations about the benefits of self-moderation tools for their well-being.
- Consider adding clear step-by-step written instructions and visual aids (eg, GIFs, screenshots, and short videos) when instructing users on the implementation of self-moderation and well-being tools.
- Consider the introduction of more default user options, such as automatically clearing search history or readjusting content level settings after a session to reduce the burden on users, giving them opt-out options.

**Platform algorithms and content allowance**

- Be explicit about what mental health–related content is allowed and restricted, using examples and unambiguous language.
- Where platforms use recommendation systems to influence content users engage with, prioritize giving users the ability to “refresh” their platform algorithm.
- Strive to enhance the robustness of automatic moderation systems, aiming for improved detection of potentially harmful mental health content (including severe, graphic, competitive, and instructive content). Recognize the complexity of this task and consider an approach that integrates human oversight.
- Consider alternatives to censoring common mental health terms, such as increasing the number of steps needed for users to access content, using overlays, and “pushing” recovery or positive mental health related content via platform algorithms.
- Consider implementing rate limits on accounts for repeated searches of potentially harmful content or algospeak terms. This could involve introducing novel help messaging or alerts after engagement attempts, which change depending on the frequency or severity of search terms.

### Early Intervention

Previous studies have shown that application onboarding periods are critical in various contexts, including assisting new users’ engagement with features [31,32], and helping patients with mental health disorders manage expectations and build confidence and proficiency with technology [33,34]. However, findings from this study showed that platforms were limited in their education of users during initial engagements, particularly lacking clear direction to “community guidelines” and “self-moderation” tools. Such a passive approach may contribute to users feeling less informed about platform features and potentially susceptible [35].

Despite this, there was some evidence of platforms using nudges—subtle prompts designed to positively influence user behavior [36]—during this period. This included TikTok’s data protection and safety videos for younger teenagers, Instagram and Tumblr’s prompts for users to follow accounts, personalizing recommendations, and Tellmi’s information regarding its limitations in terms of crisis support. These nudges served as proactive ways of engaging users with platform mechanisms, leveraging early interactions to educate or apply settings that could benefit users in the future [36], though there were some limitations, TikTok’s instructional videos could be overwhelming, especially given that online users perceive learning about privacy settings as burdensome [37]. In addition, targeting younger teenagers excluded other users who may need the guidance. Furthermore, TikTok and Instagram allowed users to skip their processes, which, although encouraging autonomy, could result in users being less informed [36]. Tumblr did not offer the same skipping option, but findings indicated that the recommendation system offered options to engage with potentially harmful content, exposing broader moderation issues [38]. This suggests that while platforms have some capacity to engage users through nudges, they may be missing a valuable opportunity for early intervention around safety features and content moderation for their respective user bases. In addition, recommendation system inadequacies need to be considered significant in the safety of new or susceptible users.

### Navigating the Platform

Instagram and TikTok offered comprehensive internal mental health–related support, including resources on suicide, self-harm, and disordered eating, as well as areas for younger or parent and guardian audiences. Integrating evidence-based resources internally and matching platform branding may be an effective approach for capturing users’ attention through familiarity and perceived reliability [39,40]. However, the volume of mental health–related resources often led to repetition, resulting in the redundancy of information and potentially user fatigue [41]. Given evidence that individuals with severe mental illness struggle to understand complex content or navigation, platforms should aim to avoid overwhelming users with excessive psychoeducation, safety, and well-being content [42,43]. Furthermore, the presence of broken links and spelling mistakes in some of these sections emphasized poor maintenance, highlighting potential concerns around the platforms’ commitment to providing high-quality, up-to-date resources [44].

Instagram also gave access to several mental health–related resources produced by other organizations, and Tellmi provided similar links to external content in various media forms. This approach allowed platforms to offer a wider range of resources, including those addressing intersectional factors in mental health, without needing to produce the content themselves, thereby improving efficiency. However, directing users away from internal platform resources may have drawbacks, including the need for monitoring external updates and possible inconsistencies in resource branding that could confuse users [44].

Following searches for harmful content, all platforms provided contact information for mental health organizations and emergency services. However, these details were often absent in other mental health–related platform spaces, missing opportunities for preventive intervention [45]. In addition, except for Tellmi, which is for young people in the United Kingdom, platforms overrepresented US resources. This reflects a broader issue of US-centric content bias on global platforms that may marginalize non–US users by limiting accessibility to relevant information [46].

Design decisions on platforms, particularly those impacting usability and accessibility to features, were also considered. Tumblr, TikTok, and Instagram used the “hamburger” icon for access to settings, policies, and resources, although some features were directly accessible during platform use. While the hamburger icon is widely recognized by experienced users, it may appear hidden or unclear to those without prior online knowledge [47]. Tellmi used a ring-bound notepad symbol for their “directory” resources page, which may be less recognizable. Research shows that users with mental health disorders often find abstract icons challenging, highlighting potential usability barriers associated with these icon designs [48].

### Encouraging User Agency

Study findings indicated that while major platforms have implemented some “self-moderation” strategies to comply with guidelines in the OSA [17], significant gaps remain in how they promote user empowerment, particularly for those engaging with mental health–related content or experiencing mental health difficulties. For instance, TikTok and Instagram introduced screen break tools to encourage user control, but these tools allowed users to return after short periods or to be easily overridden, potentially undermining their effectiveness in favor of user retention. This reinforces criticisms that commercial interests may outweigh ethical considerations in social networking design [49].

In contrast, Tumblr’s limited self-moderation tools and weak content moderation increased users’ potential exposure to harmful content, while Tellmi, although also offering fewer self-moderation tools, compensated with robust content moderation offering better avoidance from unsafe material. However, the supportive nature of interactions on Tellmi could create a sense of responsibility among users, potentially resulting in excessive engagement that current “self-moderation” tools do not address [50]. In addition, the absence of Tellmi moderators overnight could result in user migration to other platforms, leading to riskier engagements elsewhere [51]. Alternatively, TikTok’s “refresh your for you feed” feature was a novel self-moderation tool that empowered users and allowed for continual platform engagement, highlighting benefits for both users and SNSs. However, as with other self-moderation tools, its effectiveness relied on users recognizing the need for online behavior change and having the capacity to act, which may be challenging for individuals experiencing poorer mental health or with lower metacognition skills [26].

This highlights a critical limitation found across platforms—the lack of proactive intervention to raise awareness about “self-moderation” tools. These findings align with the survey by Ofcom video-sharing platform [52], which found that only 42% of UK-based video-sharing platform users were aware of platform safety mechanisms. Along with minimal prompts directing users to tools, our findings revealed insufficient education on their functionality and usefulness. There was also a significant lack of information on tool adoption or any evidence that they underwent user testing, raising questions about their efficacy. This is especially relevant considering a recent research by Bright et al [35] indicating users’ dissatisfaction with online safety technology. These findings suggest platforms may be superficially complying with OSA requirements rather than genuinely committing to empowering users. They also indicate a broader failure across the industry to address the importance of digital literacy surrounding “self-moderation” tools.

Platforms in this study also attempted to improve user safety by implementing default feature restrictions. However, default sensitivity or mature content controls often lacked clarity on whether they addressed mental health–related content or the types of content covered under each level, potentially confusing users [8]. On TikTok and Tumblr, the strictest restrictions were largely applied until users reached the age of 18 years, suggesting platforms may assume age as a qualifier of user efficacy. This overlooks the risks of removing protective measures for users who have not developed the skills or awareness needed to manage their safety independently [53]. Alternatively, Instagram, via its “teen accounts” approach allowed teenaged users to modify defaults with parental approval, encouraging a more open dialogue between users and their families. However, this approach shifts responsibility from platforms to parents. These measures also fail to address prevalent issues of age falsification among young users [54] and parents permitting their children to use social media before reaching the minimum age [52,55]. In contrast, Tellmi stood out by allowing all users to adjust their sensitivity content controls, prioritizing user agency over imposed controls [53].

Despite a lack of proactive user empowerment strategies, the changes made by platforms to improve user safety have likely been driven by the introduction of recent regulations such as the Digital Services Act in Europe [56] and the OSA in the United Kingdom [17]. These frameworks have specifically prompted platforms to implement changes that focus on the safety of young users and the moderation of dangerous or harmful content. However, the effectiveness of regulation implementation remains unclear [57,58]. For instance, an initial case study of Instagram’s “teen accounts” revealed that young people using the feature remain exposed to inappropriate content [59].

In addition, while platforms adapt to meet the technical requirements of regulations, they likely remain unable to overcome long-standing limitations in moderating harmful content without further support or guidance from legislators. Current regulations also overlook variability between types of SNS platforms, including differences in resource availability and community make-up. This lack of insight risks the introduction of regulation that may inadvertently suppress or disempower online users. Country-specific regulations further fail to account for workarounds by users, such as the use of virtual private networks, allowing them to bypass geographical restrictions and access platforms from countries where regulations do not exist. This suggests that although platforms may implement changes in response to legislation, ongoing challenges in user safety and content moderation could remain unaddressed due to gaps in regulatory focus and scope.

Our findings also showed that platforms often implied it was the user’s responsibility to identify and report harmful content, which may be problematic for susceptible users [8] and those unaware of reporting mechanisms [60]. In addition, Instagram’s claim that reported content would feature less for the reporter could discourage the behavior, as users who engage with mental health–related content may fear limiting their own access to resources or information [26]. Observations also revealed that transparency reports on harmful content removal, when available, were difficult for users to locate and interpret. Improving their visibility could alleviate user pressure by showing that much disallowed mental health–related content is automatically flagged, but the continued reliance on user reporting for content that evades moderation systems may undermine confidence.

Platforms did make efforts to streamline reporting, with Instagram and TikTok offering feedback mechanisms and Tellmi providing direct communication with human moderators. However, it remains unclear whether the reporter or reportee receives any support after harmful content is reported, and the absence of feedback or mental health resources for reporters may discourage action [8]. Furthermore, if reported users are not offered appropriate resources, platforms may inadvertently punish individuals for sharing mental health struggles, reinforcing stigma [61]. This highlights the need for greater transparency around platform reporting procedures and clearer communication about the resources and support available to those whose content is reported.

### Posting Harmful Mental Health–Related Content

All platforms gave some indication of the types of mental health content that were prohibited. However, while TikTok and Instagram included in-depth examples of allowed and disallowed content, other platforms lacked similar specificity. Furthermore, guidelines often overlooked mental health–related terminology, and there was minimal mention of rules around mental health topics unrelated to suicide, self-harm, or disordered eating. This lack of clarity could lead to subjective interpretations, resulting in self-censorship or unintentional posting of harmful material. Furthermore, only Instagram addressed the allowance of content depicting self-harm scars, a significant oversight by other platforms given that users have previously criticized moderation practices regarding this issue [26]. In addition, consequences for posting harmful content were frequently vague, with platforms using ambiguous language to describe repercussions. While this may allow for nuanced moderation, it risks confusing users and deterring them from posting around their mental health, potentially perpetuating feelings of isolation and stigma [62].

### Availability of Harmful Mental Health–Related Content

Harmful mental health content was not immediately visible on TikTok, Instagram, or Tellmi. However, on Tumblr, it was available following recommendation choices, posing higher risks to susceptible users [63]. After searching common mental health–related terms, all platforms other than Tellmi provided help resources, as expected for Tellmi as a platform focused on mental health discussion. In some instances, sensitive content overlays were used on harmful images on Instagram, which may empower users by giving them the opportunity to make informed decisions about engagement, encouraging metacognitive skills [64]. Although, if similar messages are presented repeatedly, it may result in desensitization [65] and could increase curiosity among susceptible users [26]. Furthermore, we found the application of overlays on Instagram was inconsistent, which may reduce their effectiveness.

Notably, one algospeak term resulted in content on Tellmi. Although this content was not harmful, its inclusion by human moderators indicated an acceptance of the language. This presents a broader issue in mental health content moderation where censoring common mental health terms can normalize algospeak terms as substitutes, effectively reverting platforms to their original state concerning mental health language moderation [11]. Similarly, mental health–related algospeak terms bypassed moderation on TikTok, Tumblr, and Instagram and resulted in harmful content. This highlights the danger that algospeak terms can more readily expose users to risky content, and their use instead of common mental health terms may exclude users from finding and sharing genuine information and support [12,13].

On Instagram and Tumblr, some user biographies featured the phrase “block don’t report.” This showed an online culture where users actively attempted to circumnavigate content moderation [66]. This direction to others may indicate that those engaging with mental health content believe users are responsible for their own safety through “self-moderation,” rather than relying on the platform to provide protection. It may also reflect a community norm, which could result in newer members feeling pressured to conform and avoid reporting. At the same time, the statement highlights the existence of a space where users feel empowered to express themselves by sharing content. This highlights an ongoing tension for platforms between allowing users to feel empowered through self-expression and ensuring their safety.

### Future Research

Our work found no evidence that platforms evaluated the effectiveness of their self-moderation tools and guidance relevant to user mental health and well-being. This included assessing the usability, accessibility, and function of mental health–related resources and tools as well as the acceptability and practicality of moderation procedures. Notably, there was a lack of evidence of user input and testing, potentially raising concerns about the current efficacy and value of existing measures among users. If such evaluations are being conducted internally, platforms should be encouraged to share these insights publicly to enhance transparency, adding to user confidence and supporting learning across the industry. Future research evaluating these features remains essential to ensure that platforms meet users’ needs and do not promote ineffective or even risky features or guidance.

While this study did expose gaps in the current availability and accessibility of mental health and well-being resources and functions across a selection of SNS platforms, there remains a spectrum of platforms further research should aim to investigate. This includes safety and well-being resources, features, and guidance available on social media, dating, communication, e-commerce, pornography, mental health, and gaming platforms. In addition, research is needed into newly developed SNSs such as those including artificial intelligence chatbot integration plus smaller online communities that are less likely to undergo media or governmental attention.

This study resulted in a set of preliminary recommendations for industry, which give potential ways for platforms to address mental health–related content and moderation concerns. However, these recommendations need further validation and refinement, with stakeholder input, to ensure their applicability and effectiveness.

### Limitations

Due to the rapidly evolving nature of online platforms, we acknowledge that the data collected may become outdated. This research aims to capture a moment in time, and it is possible and hopeful that platforms have since updated their practices to address some of the issues highlighted and align with our recommendations. Nevertheless, we believe these observations remain valuable in understanding user experiences. In addition, the recommendations provided are likely to be relevant for similar emerging or popular platforms.

We also understand the limitations of using the walkthrough method in this study. This approach relies on subjective interpretations of researchers’ observations, which may have led to biases. Although the checklist approach allowed the researchers to be systematic in their investigation of the platforms, it did not represent the real-world experience of a user, limiting the ecological validity. To mitigate these limitations, we included both a digital native and a digital immigrant researcher in an attempt to understand different interpretations and perspectives of platforms. However, as a next step, research should engage directly with users to reflect on their experiences of online mental health–related content moderation and safety.

Tellmi was included in this study as a smaller UK-based SNS, characterized by its user-generated content and social interaction around mental health. We recognize that Tellmi is not equivalent to Instagram, TikTok, or Tumblr in terms of target user populations, content diversity, and platform functionalities. However, given its direct focus on mental health and younger users, we considered Tellmi valuable as a comparator to platforms not specifically related to mental health in evaluating their management of user features, guidance, and resources. Through our analysis, we identified notable practice differences and approaches across all platforms that helped to refine our recommendations.

### Conclusions

These findings indicate that while platforms use some successful strategies, they are not effectively addressing the mental health moderation and well-being needs of all users. While larger platforms that undergo governmental scrutiny may introduce changes, these can seem superficial, appearing to prioritize user retention and engagement over meaningful safety intervention. Smaller platforms which face less scrutiny could also present risks due to weaker automated moderation systems resulting in access to harmful content or challenges in managing high user traffic, causing potential engagements with alternative less-moderated platforms. Using human moderation before material is posted may be the most effective approach to avoiding harmful content availability but is likely unfeasible for larger, global platforms due to scalability issues. It also raises concerns about limiting online freedoms, which could have negative consequences for users, particularly for those intentionally sharing harmful content. Platforms should focus on empowering users by educating them about safety mechanisms early, giving them agency over their decisions, and providing clear information regarding content moderation. In addition, while platforms have a responsibility toward the safety of young users, they should also consider other susceptible users, such as those with mental health disorders, during their moderation policy decisions. This study also highlights the need for improvements in the scope and global relevance of government regulations to better address user needs on platforms. Further research is essential to understand the effectiveness of existing interventions used by platforms, including self-moderation tools. User involvement is also necessary for meaningful improvement.

## Appendix

Multimedia Appendix 1 Walkthrough checklist template.

Multimedia Appendix 2 Secondary walkthrough checklist template.

Multimedia Appendix 3 Comparison table (trigger warning: description of graphic and harmful content).

## Abbreviations

OSA: Online Safety Act
SNS: social networking site
ToS: terms of service

## Data Availability

The datasets generated and analyzed during this study are available from the corresponding author on reasonable request.
