# Supplementary material for: Exploring Mental Health Content Moderation and Well-Being Tools on Social Media Platforms: Walkthrough Analysis
Source: JMIR Hum Factors. 2025 May 29;12:e69817. doi: 10.2196/69817 (PMC12163353; doi:10.2196/69817)
Supplement: Multimedia Appendix 1 [file humanfactors_v12i1e69817_app1.docx]

Moderation of Mental Health Related Content on Social Media

Walkthrough Checklist

| **App/Platform: [enter name]** |  |  | **Detailed Observations/Field Notes** | **Notes/Observations** |
| --- | --- | --- | --- | --- |
| **App Store Description** | Search app store to download specified app | Was there any information regarding moderation/safety within the app store descriptor? |  |  |
| **Open App** |  |  |  |  |
| **Signup** |  |  |  |  |
| Create a new account as a 12 year old | Go through the profile set-up steps | Were you able to setup a profile? |  |  |
| Create a new account as a 15 year old | Go through the profile set-up steps | Were you able to setup a profile? |  |  |
| Create a new account as a 19 year old | Go through the profile set-up steps | Were you able to setup a profile? |  |  |
| Continue with profile (age 19) |  | Were you prompted to read the community guidelines/ToS? |  |  |
| Terms of Service |  | How do you access them and what do they look like? |  |  |
|  |  | Is the minimum age of use stated in the ToS? |  |  |
|  |  | Did the ToS specify who moderates content on the app/platform? |  |  |
|  |  | Do the ToS specify what is moderated on the platform? |  |  |
|  |  | Did the ToS specify how mental health content is moderated on the platform? |  |  |
|  |  | How do they define and categorise mental health content? |  |  |
|  |  | Do they specify the content moderation process for content you post/publish? |  |  |
|  |  | Do the ToS specify the content moderation process for content you report? |  |  |
| Community Guidelines |  | How do you access them and what do they look like? |  |  |
|  |  | Is the minimum age of use stated in the Community Guidelines? |  |  |
|  |  | What do the Community Guidelines specify in terms of content moderation? |  |  |
|  |  | Do the Community Guidelines specify what content is moderated on the platform? |  |  |
|  |  | Did the Community Guidelines specify how mental health content is moderated on the platform? |  |  |
|  |  | How do the Community Guidelines define and categorise mental health content? |  |  |
| Privacy Policy |  | How do you access it and what does it look like? |  |  |
|  |  | What does it mention about moderation and safety? |  |  |
| Complete Signup Process |  | If you do not read the Community Guidelines/Terms of Service/Privacy Policy through your own actions, are you informed about moderation/safety during signup? |  |  |
| **Explore Content Recommendations** |  |  |  |  |
| Use the platform for 15 continuous minutes |  | Were you given instructions on how to use the app/platform? |  |  |
|  |  | Did they instruct you on any safety/reporting/self-moderation features? |  |  |
| **Searches** |  |  |  |  |
| Use search functionality for keyword ‘suicide’ |  | Were there any warnings or interventions? |  |  |
| Use search functionality for keyword ‘self-harm |  | Were there any warnings or interventions? |  |  |
| Use search functionality for keyword ‘anorexia’ |  | Were there any warnings or interventions? |  |  |
| Use search functionality for keyword ‘bulimia’ |  | Were there any warnings or interventions? |  |  |
| Use search functionality for keyword ‘eating disorder’ |  | Were there any warnings or interventions? |  |  |
| Use search functionality for keyword ‘sewerslide’ |  |  |  |  |
| Use search functionality for keyword ‘unalive’ |  |  |  |  |
| Use search functionality for keyword ‘proana’ |  |  |  |  |
| Use search functionality for keyword ‘ednotedsheeran’ |  |  |  |  |
| **Reporting Content** |  |  |  |  |
| Report content page |  | How do you access them and what do they look like? |  |  |
|  |  | What are the steps in the reporting process? |  |  |
|  |  | What are the specific steps if reporting mental health related content? [self-harm/suicide] |  |  |
|  |  | What are the specific steps if reporting mental health related content? [disordered eating] |  |  |
|  |  | How will the platform manage your report? |  |  |
| **Moderation Tools for Users** |  |  |  |  |
| Explore the tools available for users to ‘self-moderate’ | Identify the tools and use them | What tools are available on the platform, and how do you find them? |  |  |
|  |  | What are the steps to implement the tools? |  |  |
| **Moderator Safety** |  |  |  |  |
|  |  | What information is there on moderator health and safety? |  |  |
| Explore how to become a moderator |  | What additional information is there on moderator health and safety? |  |  |
| **Regional Variations** |  |  |  |  |
|  |  | Is there evidence that moderation policies and practice may be different based on geographic location? |  |  |
