# Supplementary material for: Exploring Mental Health Content Moderation and Well-Being Tools on Social Media Platforms: Walkthrough Analysis
Source: JMIR Hum Factors. 2025 May 29;12:e69817. doi: 10.2196/69817 (PMC12163353; doi:10.2196/69817)
Supplement: Multimedia Appendix 2 [file humanfactors_v12i1e69817_app2.docx]

Moderation of Mental Health Related Content on Social Media

Secondary Walkthrough Checklist

| **App: [Platform Name]** | **Action** | **Walkthrough the steps it took you to access this tool on the app.** | **From the information given in the user interface – identify the functionality and purpose of the tool/guidance.** | **Notes/Observations** |
| --- | --- | --- | --- | --- |
| **‘Self-Moderation’ Tool** | Add ‘limits’ to your account |  |  |  |
|  | Turn on ‘Break Reminder’ |  |  |  |
|  | Turn on ‘Quiet Mode’ |  |  |  |
|  | Turn on ‘Daily limit’ |  |  |  |
|  | Turn on ‘pause notifications’ |  |  |  |
|  | Snooze suggested posts |  |  |  |
|  | Add words or phrases (to be filtered) |  |  |  |
|  | Hide comments (with filtered words or phrases) |  |  |  |
|  | Hide message requests (with filtered words or phrases) |  |  |  |
|  | Control how much sensitive content you see |  |  |  |
|  | Hide like and share counts |  |  |  |
|  | Restrict an account |  |  |  |
|  | Press ‘Not Interested’ on a ‘story’ |  |  |  |
|  | Make account private |  |  |  |
|  | Add ‘supervision’ |  |  |  |
|  | Block a User |  |  |  |
|  | Restrict a User |  |  |  |
|  | Unfollow a User |  |  |  |
|  | Hide a post |  |  |  |
|  | Clear all recent searches |  |  |  |
|  | Mute Advertisers |  |  |  |
| **Reporting** | Report a user posting self-harm/suicide content |  |  |  |
|  | Report a user posting disordered eating content |  |  |  |
|  | Report content related to self-harm/suicide |  |  |  |
|  | Report content related to disordered eating |  |  |  |
| **Can you Find** |  |  |  |  |
| **From Home Page** |  |  |  |  |
